# Association between Serum Spermidine and TyG Index: Results from a Cross-Sectional Study

**DOI:** 10.3390/nu14183847

**Published:** 2022-09-17

**Authors:** Rui Zhang, Jiahui Xu, Ruixue Li, Zhecong Yu, Wei Yuan, Hanshu Gao, Wenjing Feng, Cuiying Gu, Zhaoqing Sun, Liqiang Zheng

**Affiliations:** 1 College of Public Health, Shanghai University of Medicine and Health Sciences, Shanghai 201318, China; 2 Ministry of Education-Shanghai Key Laboratory of Children’s Environmental Health, School of Public Health, Shanghai Jiao Tong University School of Medicine, 227 Chongqing South Road, Huangpu District, Shanghai 200025, China; 3 Infectious Disease Prevention and Control Center, Hohhot Center for Disease Control and Prevention, Hohhot 010070, China; 4 School of Public Health, China Medical University, Shenyang 110122, China; 5 Department of Cardiology, Shengjing Hospital of China Medical University, Shenyang 110004, China

**Keywords:** spermidine, TyG index, insulin resistance, cross-sectional study, epidemiology

## Abstract

Background: Although animal experiments have shown that spermidine (SPD) affects insulin resistance (IR), the evidence for this in humans is still scarce. We aimed to investigate the associations between serum SPD levels and the TyG index in the adult population. Methods: A cross-sectional study was carried out with 4336 participants, all of whom were adults aged 35+ years. The SPD levels in serum were detected using high performance liquid chromatography with a fluorescence detector (HPLC-FLD). The triglyceride-glucose (TyG) index was calculated as Ln [fasting triglycerides (TG) (mg/dL) × fasting glucose (mg/dL)/^2^]. Results: After multivariable adjustment, including demographic characteristics, behavioral factors associated with heath, and a history of taking medicine, SPD was inversely associated with the TyG index (β = −0.036; SE: 0.009; *p* < 0.001). Furthermore, each increase of 1 lnSPD significantly decreased the risk of IR with an odds ratio (ORs) (95% confidence intervals (CIs)) of 0.89 (0.83–0.96). Relative to the first quintile, the multivariate-adjusted ORs (95% CIs) for the third and fourth quartile group were 0.80 (0.65, 0.99) and 0.71 (0.57, 0.88), respectively. Conclusions: In conclusion, SPD was inversely associated with the TyG index. Our findings inform future exploratory research on the further mechanism of the association between spermidine and IR.

## 1. Introduction

Spermidine (SPD), a ubiquitous polycation, is associated with a variety of essential cellular functions and contributes to the maintenance of general cellular homeostasis [1]. Spermidine in tissues has three sources, namely biosynthesis, dietary intake, or production by the gut microbiota [2]. Meanwhile, SPD is a natural inducer of autophagy with many beneficial properties [3]. It has antioxidant activity, regulates enzyme function, and is required for the regulation of translation [4,5,6]. Therefore, the relationship between spermidine and health is emerging as a hot topic of research.

Previous studies elucidated that spermidine was associated with stroke [7,8], diabetes [9], obesity [10,11], metabolic syndrome [12] and other chronic diseases [13,14]; however, the results of these studies were inconsistent. Insulin resistance (IR) is defined as an insulin-mediated defect in the control of tissue glucose metabolism, initially manifested in patients with type 2 diabetes and obesity, and has been shown to be a common pathological basis for many chronic diseases [15,16]. Therefore, exploring the potential association between spermidine and IR can further clarify the mechanisms linking spermidine to various health conditions. In addition, previous animal studies elucidated that spermidine supplementation can improve IR. Ma et al. found that SPD supplementation significantly improves IR in diet-induced obese mice [10]. Moreover, a recent animal experiment showed that the oral administration of SPD attenuated IR in mice fed with a high-fat diet [17]. In addition, mice that were targeted to disrupt the SPD/spermine N1-acetyltransferase gene showed signs of insulin resistance as they aged [18]. However, the conclusions of population-based studies are not entirely consistent. A metabolomic analysis revealed that after bariatric surgery, serum SPD levels were significantly increased in morbidly obese patients and were also correlated with the remission of metabolic syndrome after surgery [12]. Furthermore, a study that included 10 IR vs. 10 insulin-sensitive (IS) non-diabetic morbidly obese Caucasian participants illustrated that SPD levels were elevated in the IR group [19]. The effect of spermidine on IR requires further investigation due to the scarce and inconsistent epidemiological evidence.

The gold standard for assessing insulin insensitivity is the hyperinsulinemic-euglycemic clamp technique. However, since the glucose clamp procedure is time-consuming and expensive, it is difficult to apply the method in large population studies and clinical settings [20]. The TyG index is a good surrogate indicator for assessing insulin resistance and is suitable for large-scale population studies [21]. Furthermore, the dietary intake of SPD obtained through food frequency questionnaires may not accurately reflect the actual concentration of SPD in vivo due to the presence of recall bias. Therefore, we chose to focus on the concentration of SPD in serum to provide a relatively objective reflection of the level of SPD in vivo on the one hand, and to provide a more convincing basis for subsequent research and promotion on the other hand. Therefore, we aimed to investigate the relationship between serum SPD levels and the TyG index in a cross-sectional study of the general Chinese population.

## 2. Materials and Methods

### 2.1. Study Population

Data were obtained from a Chinese large-scale cohort study. This cohort was originally designed as a cardiometabolic cohort to measure the association between single and clustered cardiovascular risk factors and mortality. The baseline survey was conducted in rural areas of Fuxin County, Liaoning Province, China, from June to August 2019. According to the demographic characteristics, we selected two townships in the southern part of Fuxin County and one township in its northern and eastern parts, respectively. Ultimately, thirty-three villages from these four townships were selected. By the end of 2019, the four townships had a total of 75,732 permanent residents. Participants were recruited by the government’s health department organization and underwent face-to-face centralized examinations. Participants met the following inclusion criteria: (1) ≥35 years of age; (2) ≥5 years of local residence; and (3) signed an informed consent form. Participants were excluded if they: (1) were pregnant; (2) developed severe liver and renal failure; or (3) were unwilling to take part in this study. History of severe liver and renal failure was identified with self-reported information and further confirmed by medical records. Finally, 4689 participants were recruited, which represented 6.19% of the total resident population. Written informed consent was obtained from all participants. If the participants were illiterate, we obtained written informed consent from their proxies. The procedures followed were in accordance with the ethical standards of the responsible committee for human experimentation of the China Medical University ([2018]083).

**Figure 1:**
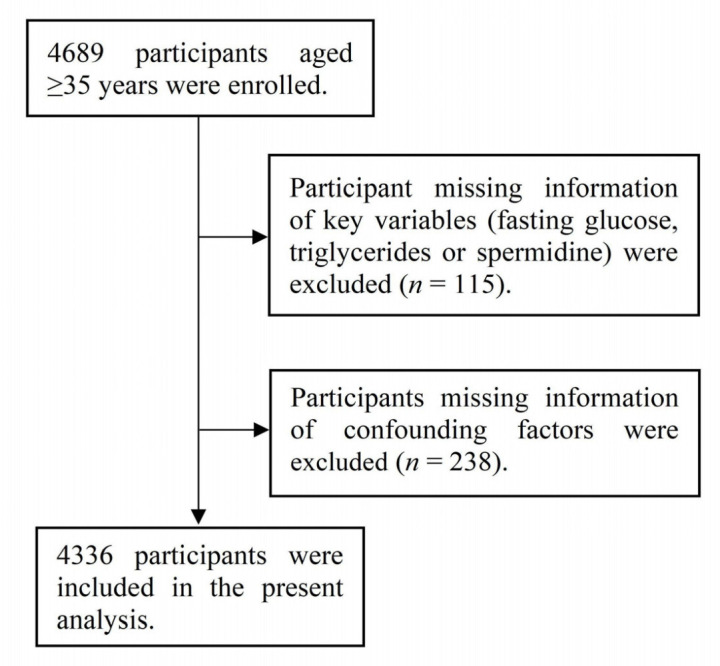
Flowchart of the participants included in the current analysis.

A flowchart of the participants included in the current analysis is presented in Figure 1. Participants with missing information for fasting blood glucose, triglycerides (TG), or SPD were excluded (*n* = 115). In addition, 238 participants were excluded due to missing information on confounders. As such, 4336 participants were included in the final analysis.

### 2.2. Measurement of Serum SPD

Participants underwent an 8 h fast prior to the examination. Antecubital vein blood samples were taken in the morning and collected in silicified vacuum glass tubes. Serum was centrifuged at 3000 rpm for 10 min and stored at −80 °C. High-performance liquid chromatography with a fluorescence detector (HPLC-FLD) was used to measure SPD levels in serum. Specifically, the main process of detecting serum SPD was as follows: SPD trihydrochloride was performed by adding 200 μL of 0.1 M HCl to 100 μL of serum. A total of 200 μL of 0.1 M HCl was added to 100 μL of serum to prepare the SPD trihydrochloride. Then, protein precipitation was performed by adding 1 mL of acetonitrile (ACN) to the mixture to precipitate the serum protein. The supernatant was blown to dryness with nitrogen at 40 °C, then 200 μL of 0.1 M HCL was added to reconstitute the dried product. Derivatization was performed by adding 200 μL of dansyl chloride and 400 μL of the buffer to the abovementioned 200 μL reconstituted solution, followed by vortexing and mixing, reacting in a water bath at 60 °C for 45 min, cooling to room temperature, adding 40 μL ammonia water, reacting in the dark for 30 min, and forming acetonitrile. A 1 mL solution was obtained, mixed thoroughly, and passed through a 0.22 μm filter. The extracted samples were analyzed using an HPLC system to obtain a standard curve. The LC column was an Agilent TC-C18 column (250 mm × 4.6 mm, 5 µm particle size). Mobile phase solutions A and B were ultrapure water and ACN, respectively. Gradient elution was selected at 0–7 min, 55–50% A; 7–25 min, 50–10% A; 25–31 min, 10% A; 31–35 min 10–55% A, and 35–40 min 55% A. The flow rate was 0.8 mL/min. The column temperature was 35 °C. Detection of wavelength was λex/λem = 340/510 nm.

### 2.3. Assessment and Definition of TyG Index

Fasting glucose in serum was measured at an accredited central laboratory with a Roche Cobas 8000 C701 automated biochemistry analyzer using the hexokinase method. TGs were measured by using a colorimeter. All laboratory equipment was calibrated prior to testing, and blinding was used to randomly code and test the blood samples from all populations. The TyG index was calculated according to the following, previously published formula [20]: TyG index = Ln [fasting TG (mg/dL) × fasting glucose (mg/dL)/2]. Additionally, the top quartile (Q4: ≥9.07) of the TyG index was defined as IR [22].

### 2.4. Assessment and Definition of Other Variables

Data on demographic variables (age, gender, and ethnicity), lifestyle factors (smoking, alcohol consumption, and physical activity), and history of medications use were collected using standardized questionnaires. Current smoking was defined as smoking at least 1 cigarette per day for 6 months. Current drinking was defined as at least 3 drinks per week for 6 months. Physical activity was classified into the following three levels: low, medium, and high. Low physical activity was defined as 30 min of moderate-intensity exercise twice a week or less, medium physical as three to four times a week, and high physical activity as more than five times a week.

Height, weight, and waistlines were measured using standardized procedures. Body mass index (BMI) was calculated as weight (kg)/height (m)^2^. According to the protocol of the American Heart Association, blood pressure (BP) was measured three times at least 1 min after a rest of at least 5 min using a standardized automated electronic blood pressure measuring device (HEM-8102A/K) [23]. Participants were asked to refrain from consuming alcohol, coffee and tea, smoking, and exercise for at least 30 min prior to BP measurement. The average of three BPs was used for the final analysis and evaluation. The history of medication use was determined by the question “have you ever taken hypoglycemic drugs or insulin/hypolipidemic drugs/antihypertensive drugs”.

We defined comorbidity as the presence of at least two chronic conditions in a predefined set of seven related chronic conditions [24]. The chronic diseases considered were coronary heart disease, stroke, diabetes mellitus, hypertension, chronic obstructive pulmonary disease, chronic hepatitis, and liver cirrhosis.

### 2.5. Statistical Analysis

Data with a normal distribution are expressed as means ± SD, SPDs with a skewed distribution are reported as medians (interquartiles), and ln was transformed to approximate normality prior to analysis. Categorical variables are represented by frequency and percentage. A one-way ANOVA, nonparametric analysis, or χ2 test was used to compare characteristics, as appropriate. The relationship between serum SPD levels and the TyG index was assessed with multiple linear regression adjusting for age, gender, ethnicity, current drinking, current smoking, physical activity, BMI, waistline, systolic BP (SBP), diastolic BP (DBP), and use of hypoglycemic drugs, or insulin, hypolipidemic drugs, and antihypertensive drugs. Multivariate logistic regression models were used to calculate the odds ratios (ORs) with 95% confidence intervals (CIs) for the associations between SPD and IR after adjusting for age, gender, ethnicity, current drinking, current smoking, physical activity, BMI, waistline, SBP, DBP and use of hypoglycemic drugs or insulin, hypolipidemic drugs, and antihypertensive drugs. We identified the potential confounders based on prior knowledge of those most commonly used in epidemiology. Prior studies suggested that age, gender, ethnicity, current smoking, current drinking, physical activity, BMI, waistline, SBP, and DBP are associated with the TyG index or insulin resistance [25,26]. We additionally adjusted the history of medication use to exclude the association of spermidine with TyG and insulin resistance related to taking medication. SPD entered the model either as a continuous variable with lnSPD or as a quartile (Q1: <13.56 ng/mL; Q2: 13.56–25.17 ng/mL; Q3: 25.17–50.49 ng/mL; Q4: ≥50.49 ng/mL), with the lowest quintile (Q1) as the reference group. Tests for trends across quartiles were performed using ordinal values in separate models. *p* values < 0.05 were considered to indicate statistical significance.

## 3. Results

The characteristics of the 4336 participants stratified by the SPD quartile are presented in Table 1. Overall, the mean (SD) age was 59.2 (9.9) years and 2786 (64.3%) of the participants were women. Gender, age, current drinking, current smoking, BMI, waistline, SBP, and TG were significantly different among the four groups.

**Figure 2:**
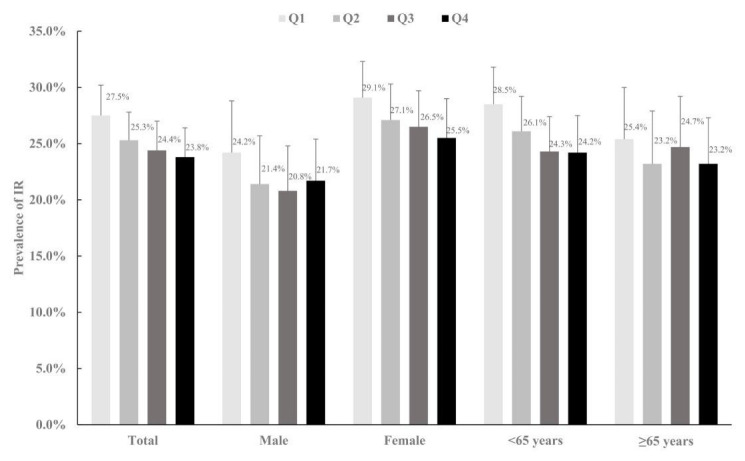
The prevalence rates (95% CIs) of IR groupings by different SPD levels. Abbreviations: IR, insulin resistance; SPD, spermidine.

The prevalence rates (95% CIs) of IR are presented in Figure 2. For participants of the first, second, third, and fourth quartile group, the prevalence of IR was 27.5%, 25.3%, 24.4%, and 23.8%, respectively.

Table 2 presents the associations between serum SPD levels and the TyG index. An Increased SPD intake led to a significantly lower TyG index (β = −0.036; SE: 0.009; *p* < 0.001) after adjusting for confounding factors, including age, gender, ethnicity, current drinking, current smoking, physical activity, BMI, waistline, SBP, DBP, and use of hypoglycemic drugs, or insulin, hypolipidemic drugs, and antihypertensive drugs. Similar trends were observed in females and participants under the age of 65, with a β (SE) of −0.044 (0.011) and −0.050 (0.012) (*p* all <0.001), respectively. We then repeated the analysis while excluding those taking antidiabetic drugs or insulin and found similar results.

Serum SPD was negatively associated with IR prevalence (Table 3). After adjusting for confounding factors, each increase of 1 lnSPD decreased the risk of IR by 11% (OR, 0.89; 95% CI, 0.83–0.96). Compared with the first quartile, the multivariate-adjusted ORs (95% CIs) for the third and fourth quartile were 0.80 (0.65, 0.99) and 0.71 (0.57, 0.88), respectively.

In the subgroup analysis, we observed an association between SPD and IR in males, females, and participants aged <60 years. In the male population, the multivariate-adjusted OR (95% CIs) for the fourth quartile was 0.67 (0.46, 0.98) compared with the first quartile. In the female population, after adjusting for confounding factors, each increase of 1 lnSPD decreased the risk of IR by 11% (OR, 0.89; 95% CI, 0.80–0.98). Compared with the first quartile, the multivariate-adjusted OR (95% CIs) for the fourth quartile was 0.73 (0.56, 0.95). In people aged younger than 60 years, after adjusting for confounding factors, each increase of 1 lnSPD decreased the risk of IR by 12% (OR, 0.88; 95% CI, 0.80–0.97). Compared with the first quartile, the multivariate-adjusted ORs (95% CIs) for the third and fourth quartile were 0.75 (0.58, 0.97) and 0.71 (0.55, 0.92), respectively. Similarly, we repeated the analysis after excluding those taking antidiabetic drugs or insulin, with consistent results. After adjusting for confounding factors, each increase of 1 lnSPD decreased the risk of IR by 12% (OR, 0.88; 95% CI, 0.81–0.96). Compared with the first quartile, the multivariate-adjusted OR (95% CIs) for the fourth quartile was 0.71 (0.56, 0.89).

## 4. Discussion

In this study, we explored the associations between serum SPD levels and the TyG index in 4336 participants based on a large-scale epidemiology survey. Serum SPD level was inversely associated with the TyG index. A higher spermidine level was associated with a lower prevalence of IR events. To our knowledge, this is the first time that serum SPD levels were shown to be associated with the TyG index among the general population.

The relationship between SPD and IR was previously proven in animal experiments [10,16,17]. Ma et al. found that daily SPD intake was inversely associated with the obesity phenotype, which provided a rationale for establishing a recommended SPD intake for obese individuals [10]. In addition, Wang et al. highlighted that for mice fed with a high-fat diet, weight loss and white adipose tissue homeostasis induced by oral spermidine may directly contribute to the resolution of IR [16]. An experiment performed on mice targeted for disruption of the spermidine/spermine N1-acetyltransferase gene supported the notion that polyamines are linked to aging-related IR [17]. A study including 32 morbidly obese patients found that patients with metabolic syndrome in remission after bariatric surgery had higher spermidine levels compared with patients who did not [12]. Our results are in agreement with the notion that high serum SPD levels are associated with a low TyG index, particularly in females and people aged younger than 60 years. The possible mechanism by which SPD reduces the TyG index is as follows: Firstly, SPD may reduce endoplasmic reticulum stress which will reduce pancreatic beta cell death by means of apoptosis [27]. Secondly, SPD may activate autophagy, thereby improving nitric oxide signaling in endothelial cells [28].

This study has several distinct strengths compared with previous studies. Firstly, differently from previous clinical studies involving dozens of patients, this is the first to explore the relationship between serum SPD levels and the TyG index in a relatively large sample (n = 4336) of the general population. Secondly, spermidine in humans has three main sources: cellular metabolism, oral absorption from dietary sources, or production by commensal gut bacteria [1]. We measured the concentration of SPD in the serum, which may help to estimate the true level of SPD in vivo.

This study also has unavoidable limitations. Firstly, we lacked an assessment of how dietary SPD intake may affect SPD levels in vivo. Thus, we were unable to establish a correlation between serum SPD levels and dietary SPD intake. Secondly, our measured serum spermidine could not accurately reflect the intracellular spermidine levels; therefore, further studies are needed to explore the exact mechanism of spermidine and insulin resistance in the future. Thirdly, we measured the level of SPD only once, and the dynamic observation of SPD changes in blood may better identify its association with the TyG index. Fourthly, we studied the rural Chinese population only, which may limit the generalizability of our study. SPD is affected by diet and intestinal flora, and the dietary habits and relevant cultural influences of different populations vary; therefore, it is meaningful to verify its consistency in different populations. Finally, because of the cross-sectional design of this study, the causality between SPD and the TyG index level was not identified. Prospective cohort studies will be conducted to further validate this finding.

## 5. Conclusions

We analyzed the relationship between SPD and the TyG index level in serum for the first time. In conclusion, SPD reflects the current TyG index level and the degree of IR. Moreover, we can expect SPD to be applied to improve IR in the future.

## Figures and Tables

**Table 1:** Basic characteristics stratified by SPD quartile ^a^.

Characteristic	Total	Q1	Q2	Q3	Q4	*p* Values
		(<13.56 ng/mL)	(13.56–25.17 ng/mL)	(25.17–50.49 ng/mL)	(≥50.49 ng/mL)	
Female, *n* (%)	2786 (64.3)	740 (68.6)	743 (68.2)	699 (64.2)	604 (56.0)	<0.001
Age, y	59.2 ± 9.9	59.2 ± 9.7	58.5 ± 9.8	59.0 ± 10.1	60.1 ± 10.1	0.001
Ethnicity, *n* (%)						0.196
Han ethnicity	2840 (65.5)	690 (63.9)	697 (64.0)	720 (66.1)	733 (67.9)	
Mongolian	1321 (30.5)	350 (32.4)	351 (32.2)	320 (29.4)	300 (27.8)	
Others	175 (4.0)	39 (3.6)	41 (3.8)	49 (4.5)	46 (4.3)	
Current drinking, *n* (%)	1306 (30.1)	294 (27.2)	308 (28.3)	327 (30.0)	377 (34.9)	<0.001
Current smoking, *n* (%)	1594 (36.8)	380 (35.2)	363 (33.3)	401 (36.8)	450 (41.7)	<0.001
Physical activity, *n* (%)						0.088
Low	1403 (32.4)	375 (34.8)	337 (30.9)	337 (30.9)	254 (32.8)	
Middle	2774 (64.0)	675 (62.6)	711 (65.3)	714 (65.6)	674 (62.5)	
High	159 (3.7)	29 (2.7)	41 (3.8)	38 (3.5)	51 (4.7)	
Hypoglycemic drugs or insulin use, *n* (%)	354 (8.2)	104 (9.6)	71 (6.5)	87 (8.0)	92 (8.5)	0.063
Hypolipidemic drugs use, *n* (%)	175 (4.0)	42 (3.9)	53 (4.9)	43 (3.9)	37 (3.4)	0.385
Antihypertensive drugs use, *n* (%)	855 (19.7)	205 (19.0)	236 (21.7)	205 (18.8)	209 (19.4)	0.307
BMI, kg/m^2^	24.7 ± 3.7	24.5 ± 3.8	24.5 ± 3.6	24.8 ± 3.7	25.2 ± 3.8	<0.001
Waistline, cm	84.2 ± 10.0	83.2 ± 9.7	83.7 ± 10.1	84.2 ± 10.1	85.8 ± 9.7	<0.001
SBP, mmHg	134.8 ± 21.5	134.0 ± 21.4	134.0 ± 21.7	134.8 ± 21.1	136.5 ± 21.7	0.025
DBP, mmHg	80.9 ± 11.2	80.3 ± 11.0	80.7 ± 11.1	81.1 ± 11.1	81.5 ± 11.6	0.067
Fasting glucose, mmol/L	6.0 ± 1.8	6.0 ± 2.2	5.9 ± 1.6	6.0 ± 1.8	5.9 ± 1.8	0.294
TG, mmol/L	1.6 ± 1.5	1.7 ± 1.9	1.7 ± 1.9	1.5 ± 1.0	1.5 ± 1.1	<0.001
SPD, median (IQR), ng/mL	25.17 (13.56, 50.48)	10.02 (8.24, 11.41)	18.70 (15.95, 21.75)	34.09 (28.80, 40.69)	78.80 (61.97, 117.73)	
TyG	8.7 ± 0.7	8.8 ± 0.7	8.7 ± 0.7	8.7 ± 0.6	8.7 ± 0.6	0.020
Comordity, *n* (%)	925 (21.3)	223 (20.7)	234 (21.5)	226 (20.8)	242 (22.4)	0.732

Abbreviations: SPD, spermidine; BMI, body mass index (calculated as weight in kilograms divided by height in square meters); SBP, systolic blood pressure; DBP, diastolic blood pressure; TG, Triglycerides. ^a^ Unless otherwise indicated, data are expressed as mean ± standard deviation.

**Table 2:** Association between serum SPD levels and TyG index ^a^.

lnSPD	Model 1		Model 2	
	β ± SE	*p*	β ± SE	*p*
Total (*n* = 4336)	−0.015 ± 0.010	0.148	−0.036 ± 0.009	<0.001
Male (*n* = 1550)	0.005 ± 0.016	0.759	−0.021 ± 0.015	0.160
Female (*n* = 2786)	−0.027 ± 0.013	0.038	−0.044 ± 0.011	<0.001
<65 years (*n* = 2902)	−0.032 ± 0.013	0.016	−0.050 ± 0.012	<0.001
≥65 years (*n* = 1434)	0.023 ± 0.016	0.147	−0.004 ± 0.014	0.772
Excluding the population taking hypoglycemic drugs or insulin				
(*n* = 3982)	−0.013 ± 0.010	0.207	−0.031 ± 0.009	0.001

Abbreviations: SPD, spermidine; SE, standard error. ^a^ Model 1 was adjusted for age and gender. Model 2 was adjusted for age, gender, ethnicity, current drinking, current smoking, physical activity, BMI, waistline, SBP, DBP, and use of hypoglycemic drugs or insulin, hypolipidemic drugs, and antihypertensive drugs.

**Table 3:** Association between serum SPD levels and IR ^a^.

	lnSPD	Q1	Q2	Q3	Q4	*p* Value for Trend
**Total, OR (95% CI)**						
Model 1	0.96 (0.90, 1.03)	1.00 (Ref.)	0.89 (0.74, 1.08)	0.86 (0.71, 1.05)	0.86 (0.70, 1.03)	0.090
Model 2	0.89 (0.83, 0.96)	1.00 (Ref.)	0.91 (0.73, 1.12)	0.80 (0.65, 0.99)	0.71 (0.57, 0.88)	0.001
**Male, OR (95% CI)**						
Model 1	0.98 (0.87, 1.10)	1.00 (Ref.)	0.87 (0.62, 1.22)	0.92 (0.65, 1.28)	0.86 (0.61, 1.22)	0.483
Model 2	0.91 (0.80, 1.03)	1.00 (Ref.)	0.85 (0.59, 1.24)	0.86 (0.60, 1.25)	0.67 (0.46, 0.98)	0.054
**Female, OR (95% CI)**						
Model 1	0.95 (0.87,1.04)	1.00 (Ref.)	1.02 (0.81, 1.29)	0.87 (0.69, 1.11)	0.86 (0.68, 1.10)	0.123
Model 2	0.89 (0.80, 0.98)	1.00 (Ref.)	0.99 (0.77, 1.28)	0.81 (0.62, 1.05)	0.73 (0.56, 0.95)	0.007
**<65 years, OR (95% CI)**						
Model 1	0.94 (0.86, 1.03)	1.00 (Ref.)	0.95 (0.75, 1.19)	0.78 (0.61, 0.98)	0.81 (0.64, 1.03)	0.029
Model 2	0.88 (0.80, 0.97)	1.00 (Ref.)	0.97 (0.75, 1.24)	0.75 (0.58, 0.97)	0.71 (0.55, 0.92)	0.002
**≥65 years, OR (95% CI)**						
Model 1	1.03 (0.91, 1.16)	1.00 (Ref.)	0.88 (0.62, 1.25)	1.03 (0.73, 1.45)	1.04 (0.74, 1.48)	0.628
Model 2	0.93 (0.82, 1.07)	1.00 (Ref.)	0.86 (0.58, 1.26)	0.91 (0.62, 1.33)	0.79 (0.54, 1.16)	0.299
**Excluding the population taking hypoglycemic drugs or insulin, OR (95% CI)**						
Model 1	0.94 (0.87,1.01)	1.00 (Ref.)	1.02 (0.82, 1.25)	0.91 (0.73, 1.12)	0.83 (0.67, 1.04)	0.061
Model 2	0.88 (0.81, 0.96)	1.00 (Ref.)	0.98 (0.79, 1.23)	0.84 (0.67, 1.05)	0.71 (0.56, 0.89)	0.001

Abbreviations: SPD, spermidine; OR, odds ratio; CI, confidence interval. ^a^ ORs and CIs were calculated using logistic regression models. Model 1 was adjusted for age and gender. Model 2 was adjusted for age, gender, ethnicity, current drinking, current smoking, physical activity, BMI, waistline, SBP, DBP, and use of hypoglycemic drugs, or insulin, hypolipidemic drugs, and antihypertensive drugs.

## Data Availability

The data that support the findings of this study are available from the corresponding authors; however, restrictions apply to the availability of these data, which were used under license for the current study, and so are not publicly available.
